# DNA Damage and Chromatin Conformation Changes Confer Nonhost Resistance: A Hypothesis Based on Effects of Anti-cancer Agents on Plant Defense Responses

**DOI:** 10.3389/fpls.2018.01056

**Published:** 2018-07-24

**Authors:** Lee A. Hadwiger, Kiwamu Tanaka

**Affiliations:** Department of Plant Pathology, Washington State University, Pullman, WA, United States

**Keywords:** nonhost resistance, DNA damage, DNA conformation, chromatin structural changes, anti-cancer agents

## Abstract

Over the last decades, medical research has utilized DNA altering procedures in cancer treatments with the objective of killing cells or suppressing cell proliferation. Simultaneous research related to enhancing disease resistance in plants reported that alterations in DNA can enhance defense responses. These two opposite perspectives have in common their effects on the center for gene transcription, the nuclear chromatin. A review of selected research from both anticancer- and plant defense-related research provides examples of some specific DNA altering actions: DNA helical distortion, DNA intercalation, DNA base substitution, DNA single cleavage by DNases, DNA alkylation/methylation, and DNA binding/exclusion. The actions of the pertinent agents are compared, and their proposed modes of action are described in this study. Many of the DNA specific agents affecting resistance responses in plants, e.g., the model system using pea endocarp tissue, are indeed anticancer agents. The tumor cell death or growth suppression in cancer cells following high level treatments may be accompanied with chromatin distortions. Likewise, in plants, DNA-specific agents activate enhanced expression of many genes including defense genes, probably due to the chromatin alterations resulting from the agents. Here, we propose a hypothesis that DNA damage and chromatin structural changes are central mechanisms in initiating defense gene transcription during the nonhost resistance response in plants.

## Introduction

Features of DNA-specific agents and their actions on cancer cells may share modes of action related to those inducing disease resistance in plants. The objective of cancer treatments is mainly to selectively stop cancer growth with little collateral damage to healthy cells. Some of the same DNA-specific compounds (Hendry et al., 2007) have been shown to activate defense response genes, termed pathogenesis-related (PR) genes (Hartney et al., 2007; Hadwiger, 2009). Research on the plant side is aimed at stopping fungal growth. The characterization of DNA damage-induced protein synthesis in plants is variable and involves traits ranging from DNA damage-related repair proteins to defensins (peptides) that are directly toxic to fungal pathogens (Chiang and Hadwiger, 1991; Almeida et al., 2006).

## Hypothesis Defined

Pathogenesis-related genes are major contributors to the plant’s nonhost resistance to pathogens (Hadwiger, 2015a). In addition, the DNA-specific signals for activation of these genes can be initiated by “elicitors” or “effectors” of pathogen origin (Jones and Dangl, 2006; Boller and Felix, 2009). The transcription of these defense genes is ultimately coded by the DNA within the chromatin of the nucleus. Based primarily on the accumulated data on defense gene activation in pea endocarp tissue we are hypothesizing that multiple DNA-specific agents can activate PR genes and stimulate secondary metabolic pathways (e.g., producing antifungal compounds called phytoalexins) by generating direct effects on chromatin conformation. In a manner similar to how effectors can initiate signals (via cascading routes) to engage the transcription factors and positively affect stalled genes, *the DNA/chromatin-specific agents can increase transcription via direct conformational changes* (Hadwiger, 2008).

This paper assembles mechanistic information from current and previously published literature on transcription initiation (Hager et al., 2009). Because of the complexity of chromatin, the understanding of its ability to determine how and when the appropriate genes within are suppressed or expressed, is a challenge for all eukaryotic research. The RNA polymerase complex that transcribes the DNA code is confronted by a tightly packed genomic DNA in a nucleosome structure. Thus, gene transcription requires that a single DNA strand transit the DNA polymerase II enzyme in an environment of tight DNA helixes and attached nuclear proteins (Ma et al., 2013). Transcription benefits from removal of DNA helices and temporarily dissociating DNA from histones and other nuclear proteins (Yaniv, 2014). The genes coding PR and other defense gene products are apparently silent, stalled or partially suppressed prior to contact with a fungal pathogen. The suppressed environment of sensitive DNA regions (Teves and Henikoff, 2014) can be changed by: DNA intercalators, DNA base substitution, thymidine dimerization, DNA minor groove insertion, histone modification or removal, DNA strand cleavage and other chromatin-specific effects– to a transcription positive state. Within these agent actions are the eliciting agents, chitosan oligomers (Kendra et al., 1989) and a single strand cleaving DNase known to be released by pathogens (Hadwiger and Polashock, 2013) and transferred to the host nucleus in the pea nonhost resistance response.

Our hypothesis is that these general conformational changes occur within sensitive regions present in multiple chromosomes since the genomic mapping of the pea genome locates PR genes in multiple chromosomes (Pilet-Nayel et al., 2002; Ramirez-Prado et al., 2018). We also realize that DNA/chromatin changes can also stimulate some genes not directly involved in disease resistance. The following paragraphs detail the data upon which the hypothesis was derived.

## DNA Damage: Insights Into the DNA Targets of Anticancer Agents and Phytoalexin Elicitors

Specific DNA altering actions including DNA intercalation, DNA distortion, DNA base substitution, DNA single and double strand cleavage, alkylation and methylation, DNA binding and exclusion in cancer related research (Martinez and Cha′con-Garcia, 2005) compare with the action of many of the same agents affecting resistance responses investigated primarily in the model endocarp tissue system of pea plants (*Pisum sativum*) (Hadwiger, 2015a). Early research on disease resistance in pea tissue revealed alterations in nuclear DNA that enhance defense responses (Hadwiger and Schwochau, 1971). These two opposite perspectives have in common their effects on the center for gene transcription, nuclear chromatin (Nair and Kumar, 2012). The similarities of action at the chromatin level in both systems are based on the degree of interaction.

The chromatin/DNA perspective presented herein by-passes a different interpretation of the signaling events that involve the plant receptor-like kinases as initiators of disease resistance or plant defense that is reviewed elsewhere (Nürnberger et al., 2004; Boller and Felix, 2009; Antolin-Llovera et al., 2014). Briefly, such signaling between an elicitor PAMPs (pathogen-associated molecular patterns) via receptor-mediated transfer to specific defense response genes within chromatin or intact pea tissue has been observed but primarily with high levels of two PAMPs (Hadwiger and Chang, 2015). These high PAMP concentrations were also associated with DNA damage and thus have commonality with the DNA-specific agents discussed herein.

Cell death or suppression in cancer following high-intensity treatments may be accompanied by chromatin distortions capable of activating the expression of less-desirable collateral genes. Likewise, in the pea endocarp, high-level treatments of DNA-specific agents can cause cell death, while low-level chromatin alterations activate the defense genes associated with immunity, i.e., nonhost resistance (Hadwiger et al., 1974; Choi et al., 2001; Hartney et al., 2007; Isaac et al., 2009). Some of the anticancer drugs remaining in use today are DNA damaging agents, and those that have been used to the best advantage in the past are being re-visited (Gurova, 2009). These agents have the potential to target the DNA of tumor cells, resulting in their destruction. However, their clinical use can result in adverse side effects, and since some are also carcinogenic, their continued use can promote secondary cancers.

## DNA Damage, Damage Repair, and Chromatin Alterations in Cancer and Age-Related Diseases of Humans

DNA repair contributes to innate and acquired immunity (Song et al., 2014). DNA damage triggers the activation of DNA repair pathways and DNA repair protects against oxidized DNA damage generated by infectious and inflammatory diseases. Thus, DNA damage is involved in innate and adaptive immunity (Fontes et al., 2014). At the transcriptional level there is the regulation of cytokines and other genes involved in the inflammatory response. Chemical modifications to DNA and the histone components of chromatin potentiate gene expression. As an example, chromatin must become accessible to allow activation-induced cytidine deaminase (AID)-mediated deamination of cytosines in DNA (Daniel and Nussenzweig, 2013). In response to DNA damage there is a removal of DNA lesions. In the arousal of the immune system there can be an expression of antimicrobial peptides and development of ligands for receptors found on immune cells. Components that can arouse include DNA damage sensors, transducer kinases, and effectors (Nakad and Schumacher, 2016). Some progress has been reported in distinguishing which molecular and cellular pathways of the DNA damage activate immune signaling (Kastan and Bartek, 2001).

## Induction of Pea Defense Responses

**FIGURE 1:**
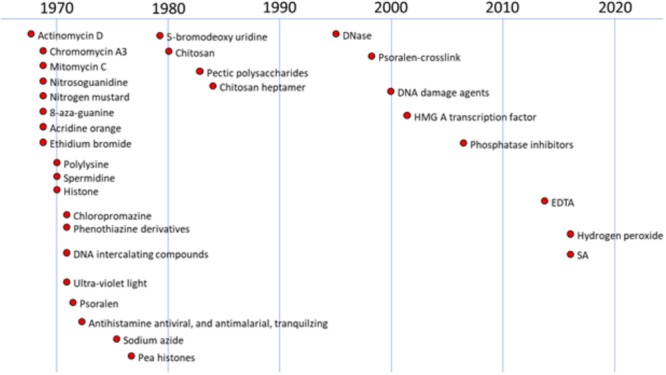
History of studies regarding anti-cancer and other compounds on DNA damage in plants. The figure were created based on the following references (chronological order): Schwochau and Hadwiger, 1968, 1969; Hadwiger and Schwochau, 1970, 1971; Hadwiger and Martin, 1971; Hess and Hadwiger, 1971; Hadwiger, 1972a,b; Hadwiger et al., 1976, 1977, 1995; Sander and Hadwiger, 1979; Hadwiger and Beckman, 1980; Walker-Simmons et al., 1983; Kendra et al., 1989; Parsons and Hadwiger, 1998; Choi et al., 2001; Klosterman et al., 2003; Hartney et al., 2007; Hadwiger and Tanaka, 2015; Hadwiger and Tanaka, 2017b; Tanaka and Hadwiger, 2017.

Investigations into the induction of plant defense responses by DNA-specific compounds in peas have occurred in parallel over multiple decades (**Figure 1**). Messenger RNA from pea tissue treated with DNA-specific agents was subsequently translated *in vitro.* This technique identified the total array of newly expressed gene products as characteristic protein patterns in 2-D electrophoretic separations. These patterns enhanced by the DNA specific anti-cancer actinomycin D in the plant host were similar to those induced following inoculation with fungal pathogens (Loschke et al., 1983). Both treatments also promoted the production of the anti-fungal phytoalexin, pisatin (Schwochau and Hadwiger, 1968; Hartney et al., 2007; Hadwiger and Tanaka, 2017a).

**FIGURE 2:**
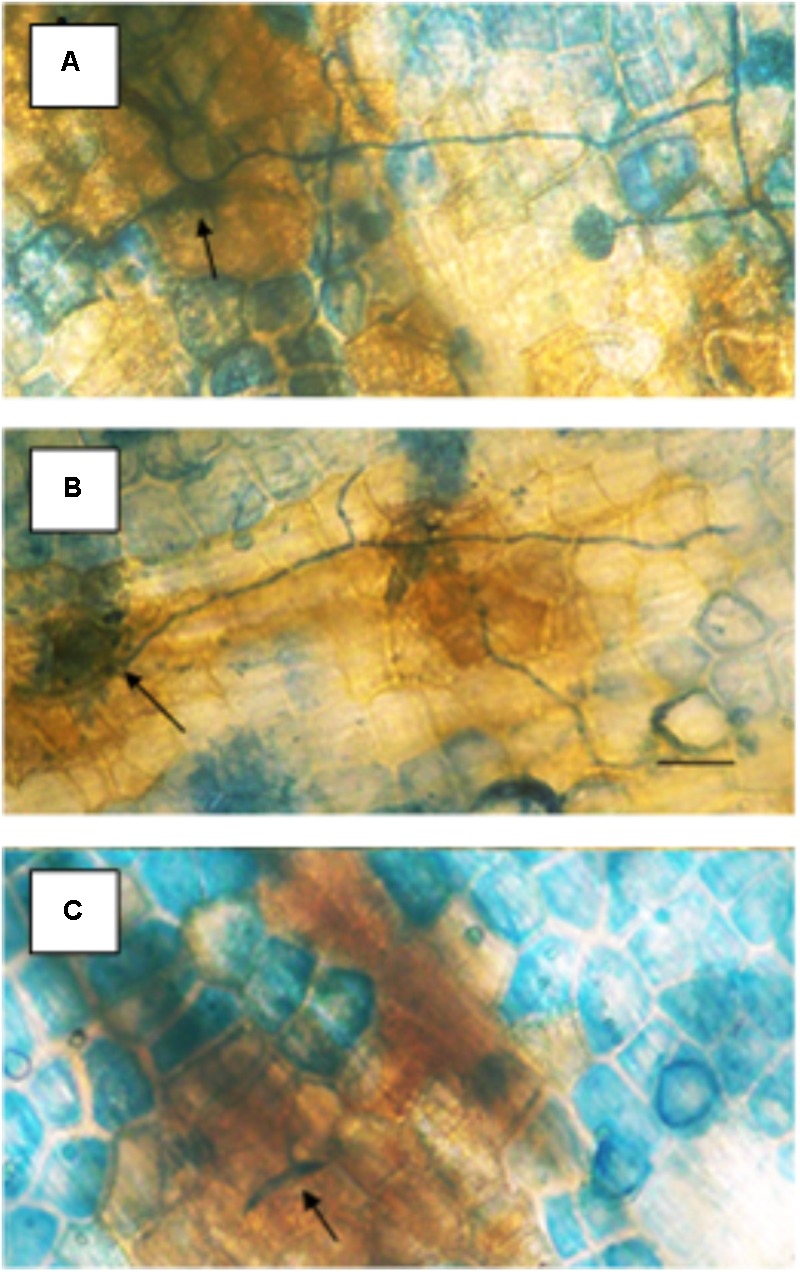
Effect of different concentrations of actinomycin D applied 1 h prior to inoculation on the susceptibility of pea endocarp tissue to the true pathogen, *Fusarium solani* f. sp. *pisi* (Fspi). The resistance responses of pea endocarp tissue against a pea pathogen (Fspi) are sharply influenced by different concentrations of actinomycin D applied 1 h prior to inoculation. Concentrations in photos are as follows: **A** = H_2_O; **B** = 1 μg/ml; and **C** = 3 μg/ml. Arrows indicate the inoculated spore. Bar = 50 microns.

Specific concentrations of actinomycin provided resistance against *Fusarium solani* f. sp. *pisi* (Fspi) a true pathogen in pea (**Figure 2**). The variation of resistance that is concentration-related, probably due to the progression of DNA changes as more actinomycin molecules become involved. Actinomycin D 1 μg/ml applied 1 h prior to the pathogen spores (**Figure 2B**) there gave no cytologically detectible induction of resistance allowing the pathogen to proceed as it did following the water treatment (**Figure 2A**) in the absence of the hypersensitive host response. At 3 μg/ml (**Figure 2C**) the presence of actinomycin induces a resistance that is a plant disease resistance response rather than a direct antifungal action.

Follow-up research utilized the chemical properties of other DNA-specific agents to investigate the basis of defense gene induction in plants, which may relate to DNA conformations or chromatin alterations.

The actions of compounds such as actinomycin D that specifically target DNA base sequences were valued for use because of the available background of physical and chemical information. The DNA intercalating property was first thought to primarily inhibit RNA synthesis; however, there were reports that it super-induced certain genes in other eukaryotes (Steinberg et al., 1975). Actinomycin D was also found to increase mRNA for specific pea genes. Examination of chromatin spreads from pea cells injected with labeled uridine indicated that regions of the chromatin are unraveled by actinomycin D, and unraveled chromatin supports hot spots of RNA synthesis (Hadwiger, 2015a). The action of actinomycin D demonstrates the complexity of DNA damage-related changes. The defense response induction by DNA-specific agents in plants was obtained with low actinomycin concentration levels. Actinomycin D was widely utilized in biological research for its ability to complex intimately (Reich and Goldberg, 1964) with DNA by intercalating the planer ring structure between base pairs and subsequently suppressing mRNA production (Flamm et al., 1966). It was noted that the binding of actinomycin D to the DNA in chromatin was restricted by the chromosomal proteins, and thus the binding of actinomycin D to chromatin could be a measure of the amount of DNA not masked by the chromosomal proteins (Beato et al., 1970). Alternately, this measure was used in plant systems to determine how much externally applied actinomycin D was transferred to the nucleus and to evaluate the open regions of DNA in pea cells; and the increased template activity that developed following treatment with elicitors and fungal challenges (Hadwiger et al., 1974).

**FIGURE 3:**
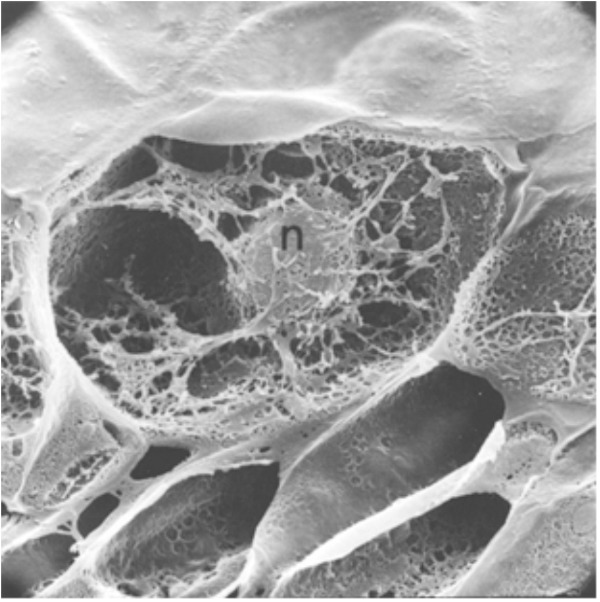
A cross-section of a pea endocarp cell, viewed in a scanning electron microscope, showing the intimate association of the pea nucleus with the cytoskeleton. Reproduced from a previous publication (Hadwiger and Adams, 1978).

These results demonstrated that both anticancer agents and defense gene activators can influence the structure and function of chromatin. Why is actinomycin D not inhibiting RNA synthesis in pea? In bacterial cells, actinomycin D is able to intercalate DNA at a rate of 1 molecule per 1000 base pairs and successfully suppress mRNA production (Hyman and Davidson, 1970). Alternately, the optimal induction of pea defense responses occurs when less than 1 molecule of actinomycin D inserts per 10,000 DNA base pairs (Hadwiger et al., 1974), a level that does not significantly suppress RNA synthesis. Thus, there is an apparent difference in action between plants and other systems based on the degree of intercalation. The activation of defense genes in pea tissue is proposed to occur by direct action on chromatin structure (Isaac et al., 2009) and is often accompanied by DNA damage. This disruption can be observed by electron microscopy (Hadwiger and Adams, 1978). The regions of disrupted chromatin structure have been shown to be regions of intense labeling with RNA precursors (Hadwiger, 2015a). The chromatin alteration hypothesis has been further tested in pea endocarp tissue and is compared with a series of compounds with well-researched modes of action (Hartney et al., 2007). Chromosome dynamics can also be influenced by inherent cytoskeleton polymers such as actin filaments, microtubules and intermediate filaments that connect to the nuclear envelope (**Figure 3**) (Spichal and Gabre, 2017). The smaller of these molecules can enter the nucleus and act as chromatin remodelers.

In pea, an assay for detecting agents initiating the transcription of defense responses monitors a secondary pathway that culminates in part with the production of the anti-fungal isoflavonoid, pisatin. Compounds that are elicitor-positive in this assay were further examined to determine whether similar changes occur in the elicitation of total disease resistance in pea by a bean pathogen (nonhost resistance) or in furthering susceptibility to both pea and bean-specific pathogens (Hartney et al., 2007). Additional assays of pea tissue involved cell fractionation and cytological preparations that specifically examined DNA damage (Isaac et al., 2009), nuclear protein modification (Klosterman et al., 2003), and nuclear diameters changes (Tanaka and Hadwiger, 2017). As indicated, the accumulation of phytoalexin, pisatin, and PR gene activation are responses that are associated with the defense response of pea.

## Variation in DNA-Specific Agents Action

The modes of action of selected compounds on DNA *in vitro* are defined in **Table 1** and their relative effects on the accumulations of the phytoalexin, pisatin are presented in **Table 2**. A large number of cyclic molecules have the potential to intercalate between the base pairs of DNA. Many derivatives of acridine have been shown to positively induce pisatin production (Hadwiger, 1972a). This action is shared by the compounds with planar three ring structures (e.g., in ethidium bromide). A positively charged nitrogen in the azole ring or on the side chain presumably attracts the negatively charged phosphate groups of DNA (Schwochau and Hadwiger, 1968). Unfortunately, many medically important compounds, including antihistamines, antimalarials, decongestants, chelators, etc., are also capable of intercalating DNA (Hadwiger, 1972a). Not all DNA intercalators are cytotoxic. Some small molecule drugs have now been shown to have a wide range of biological activities: i.e., vitamins, hormones, hormone antagonists, antipsychotics, antidepressants, and antihistamines. The DNA helix is flexible and can be readily wound or unwound. When unwound cavities appear between the base pairs, the space approximates that of small molecule natural products. For example, the shape of the steroid hormone estradiol is a good fit between base pairs of unwound DNA (Hendry et al., 2007). The plant hormone gibberellic acid fits into the intercalation site 5′-dTdG-3′ 5′-dTdA-3′ (Witham et al., 1978). Other natural products, such as caffeine, vitamin D and riboflavin, fit into unwound DNA (Hendry et al., 1977). The specific sequences in DNA into which ligands best intercalated were found in the consensus sequences of genes activated by nuclear receptors, indicating that intercalation was central to their mode of action.

**Table 1:** Action modes of some DNA-specific agents.

DNA specific agent	DNA affinity/sequence specificity/action mode	Reference
Mithramycin	GC-rich seq.- displaces Sp1 transcription factor, minor groove binding	Barcelo et al., 2010
Ethidium bromide	DNA intercalator	Lenglet and David-Cordonnier, 2010
Acrid. orange	DNA intercalator, DNA single strand binder	Lenglet and David-Cordonnier, 2010
Chitosan	Chitosan heptamer fits in DNA minor groove	Hadwiger and Beckman, 1980
Distamycin A	Inhibitor of helicase and topoisomerase I-II, minor groove binder, stimulates Pol II pause site	Varqiu et al., 2008; Nelson et al., 2007
Neomycin	Stabilizes DNA triplex TAT	Willis and Arya, 2006
Daunomycin	Intercalates Adj.G/C bp on 5’side of A/T bp; Induces DNA unwind; Evicts histone from minor groove	Quigley et al., 1980
Spermine	A-DNA backbone bridging major and minor grooves	Bryson and Greenall, 2000
Hoechst 33258	AT tract-topoisomerase poison; DNA minor groove binding and intercalates DNA bases	Miskovic et al., 2013
DAPI	AT-specific; minor groove binding; not topo I poison	Miskovic et al., 2013

**Table 2:** Pisatin production in pea endocarp tissue 24 h after treatment with DNA-specific compounds, capable of DNA intercalation or minor groove localization.

Agent appl. mg/mL ->	1.0	0.5	0.25	0.12	0.06	0.03	0.015
Mithramycin	258.5	209.6	264.9	283.8	146.0	3.2	0.0
Ethidium br.	18.2	43.1	130.9	104.9	97.2	131.7	131.6
Acrid. orange	104.3	14.9	9.7	9.8	9.0	6.0	8.0
Chitosan hep.	50.4	95.9	8.4	25.2	19.4	7.6	–
Distamycin A	73.3	40.5	30.3	22.9	14.3	6.9	3.7
Neomycin	62.1	5.2	0.0	0.0	0.0	0.1	0.0
Daunomycin	44.5	44.3	52.1	52.2	4.7	4.9	2.1
Spermine	22.9	37.3	15.6	17.5	5.6	9.5	–
Hoechst33258	24.1	14.3	17.8	8.0	0.0	13.9	0.0
DAPI	10.5	7.5	8.9	8.4	4.7	4.3	5.5

*Pisatin (μg/g fresh weight) produced by pea endocarp tissue in 24 h following the application (25 μL/pod half) of the respective concentrations of DNA-specific agents were measured. Pisatin was extracted and analyzed by protocol (Hadwiger and Tanaka, 2017a). Values represent the average of two extractions. Water treated tissues produced no detectible pisatin spectra and were used to develop a baseline of 309 nm absorbance. Average of two replications. The variance in range between replicate values did not exceed 20%.*

**FIGURE 4:**
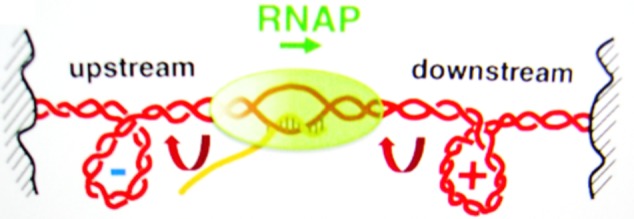
The supercoiling of DNA is a removable barrier to the RNA polymerase complex (RNAP) transcription of genes. Reproduced with copyright permission (Ma et al., 2013).

The intercalator modes of action are also likely to occur by altering the DNA torsions (unwinding) that can affect the transcription of some genes (Ma et al., 2013). The mechanics by which transcription is affected by DNA intercalators have been investigated. Although there are multiple interpretations, the following actions and conditions are well understood (Pruss and Drlica, 1989):

The packaging of DNA into the cell is assisted by histones and supercoiling, often causing negative supercoiling of the DNA. The supercoiling of the DNA in advance of the polymerase transcription complex must be removed, and the polymerase action itself is accompanied by supercoiling (**Figure 4**). As the region in front of the polymerase is unwound, there is compensatory positive supercoiling well ahead of the complex (Gilbert and Allan, 2014). Alternately, the DNA behind the complex is rewound with the development of compensating negative supercoils. DNA intercalators can twist DNA, thus affecting the supercoiling independent of the aid from a protein. Topoisomerases and DNA gyrases can relieve some of the stress. Some SWI/SNF genes code for gyrase enzymes. Additionally, SWI/SNF complexes can cause a bulge mechanism that may cause the dissociation of DNA at the edge of the nucleosome, followed by re-association of the DNA inside the nucleosome (Tang et al., 2010). Such complexes can function as tumor suppressors.

As indicated earlier, extensive research on the DNA-specific intercalator actinomycin D indicates the diversity of action *in vivo*. Actinomycin D was found to be a super inducer of the synthesis of some animal genes (Chatterjee et al., 1979). In an early screening of intercalators, we found actinomycin D and other intercalators to be strong activators of plant defense responses (Schwochau and Hadwiger, 1968). Actinomycin D action in pea tissue, in contrast to mRNA inhibition, has been explained in various ways, such as suppression of the production of transcription factors or suppression of RNase activity. However, as a general conclusion of the action of DNA intercalators in pea tissue, we propose that the torsional effect on the DNA helix is a major factor in promoting transcription, as indicated in cancer research (Teves and Henikoff, 2014). Additionally, because of the many similarities of plant and animal chromatin structure and the effect of such DNA-specific compounds on plant chromatin, concentrations below the lethal action are likely acting on varying levels of transcription enhancement and thus on the *differential* activation of genes.

## Substitution of DNA Bases and Helixes

Externally applied base analogs, such as 5-bromo deoxyuridine and 5-iododeoxyuridine, can activate the pisatin pathway in pea (Sander and Hadwiger, 1979). The base analog must be incorporated into pea DNA before any induction occurs. The nuclei undergo condensation just prior to the detection of the induced increase in phenylalanine ammonia lyase (PAL) activity. The mode of action involved the insertion of a base analog into the DNA, and the transcriptional increase was likely due to a change in the DNA helical structure during the removal of the aberrant abduct.

## DNA Cross-Linking Agents

The alteration of the DNA helix that developed from a cross-linked psoralen activates phytoalexin (pisatin) production in pea endocarp tissue and is likely triggered during the DNA repair process that would remove this aberration (Parsons and Hadwiger, 1998). In humans, such cross-linking may be general along the genome as the associated symptoms are extensive. The effect of cross-linking DNA by psoralen compounds was first reported as an environmental hazard on celery harvesters. Psoralen compounds develop when celery plants are infected by the fungal pathogen *Sclerotinia sclerotiorum* (Floss et al., 1969). The hazardous effect on workers occurred when the psoralen entered the skin of their hands. The additional environmental action came from the UV content of sunlight that enables the compound to cross-link DNA strands. The psoralen is activated to form covalent bonds. The subsequent human symptom was tumorous growth on the workers’ hands. Psoralen compounds have also been shown to activate multiple plant defense responses in pea endocarp tissue (Parsons and Hadwiger, 1998). Prior to the development of other molecular assays, the psoralen cross-linkage was also utilized to locate DNA segments within open reading frames of the PR genes, which provided evidence that the DNA abduct had occurred in the vicinity of the defense gene. This site-specific adduct was detected on southern blotting analyses run on alkaline gels (the cross-link of DNA slowed the electrophoretic of cross-linked segments and not the migration of alkali separated DNA segments). The precise effect of crosslinking in activating the pea defense response is not known; however, the removal of this adduct, such as the removal of other adducts, renders the DNA free to unwind or modify as the repair is undertaken.

**FIGURE 5:**
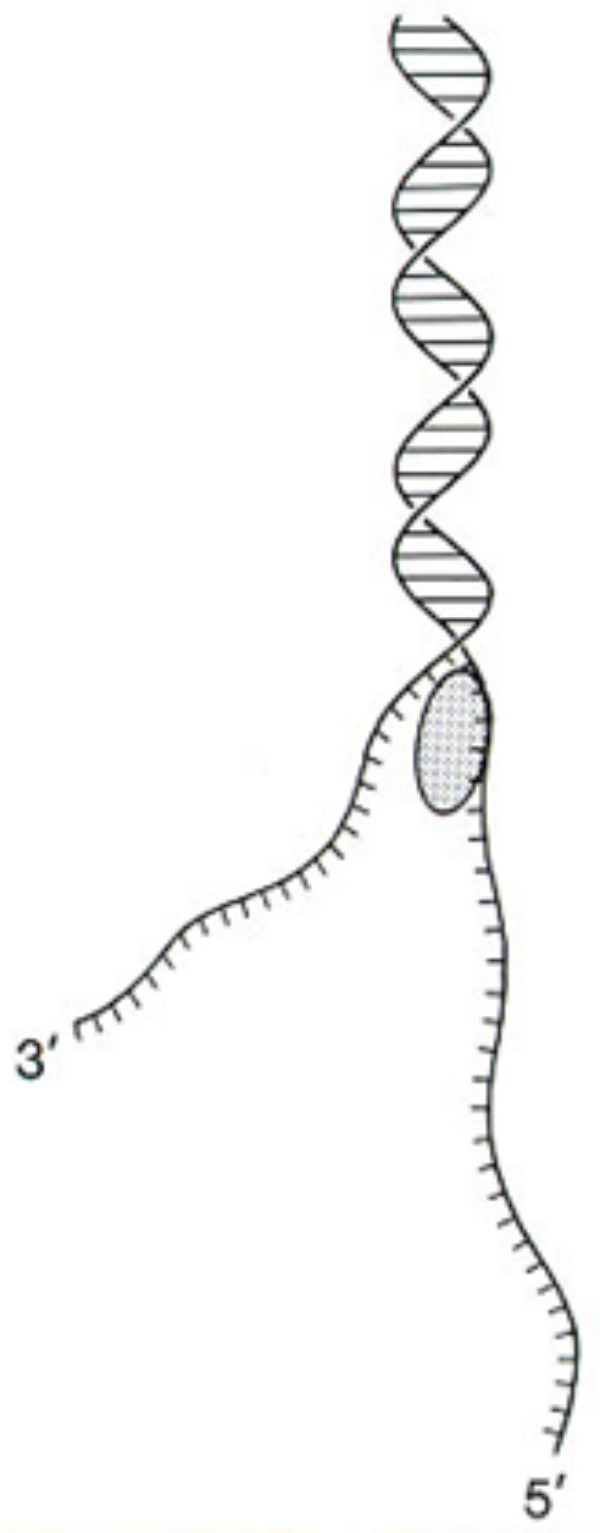
DNA single strands can develop during DNA repair or by DNase I-like or gyrase-like enzymes. Reproduced from a previous publication (Neigeborn and Carlson, 1984).

The DNA within chromatin can be negatively or positively helically coiled; thus, the presence of these supercoils can be obstructive to the progression of the RNA polymerase complex. The progression of this complex along the DNA molecule during transcription requires an absence of obstruction, as well as a separation of the strands, as shown in the drawing (**Figure 5**). The loosening of the nucleosome structure by a single strand cleaving DNase can occur both by freeing a single strand and exposing DNA for enzyme access and by allowing a release of the negative helix of the supercoiled DNA.

## Biotic DNA Targeting Agents

Fungus-related DNase function, in support of the growing fungal mycelium, was thought to occur as a means to break down DNA as a nutritional source of nucleic acid bases. A fungal DNase capable of cleaving single DNA strands is synthesized in most fungi (Hadwiger and Polashock, 2013) with an N-terminal signal peptide that enables it to cross membranes (Klosterman et al., 2001). However, as an inadvertent occurrence, the immediate plant defense response slows fungal growth. The resultant DNase accumulation that normally occurs in old mycelia for the purpose of digesting and recovering DNA components for reuse now occurs in the hyphal tip. It appears to accumulate close to the growing tip and effectively cleaves the single strands of the DNA that must remain intact for cell division. In the absence of a functional nucleus, fungal growth is terminated (Hadwiger, 2015c). All of the genomes of fungi sequenced thus far contain the DNA coding sequence for this mitochondrial DNase (Hadwiger and Polashock, 2013). The universality of the DNA strand cleaving function in eliciting a defense response is likely a major contribution to the development of “nonhost resistance” that protects plants from all but their true pathogens. The growth of a true pathogen is not so severely suppressed by the pea plant defense response, and mycelial tips can retain some viable nuclei. Mycelia with viable nuclei can continue growth on the plant tissue (Hadwiger, 2015c), as the major defense response subsides.

Naturally occurring proteins/peptides and synthesized polymers rich in the basic amino acids arginine (A) and lysine (K) were found to be capable of producing pisatin in peas. Protamine, histones, spermidine, spermine and some basic enzyme protein domains present in RNase and snake venom elicit pisatin production. All are rich in basic amino acids or basic charges (Hadwiger and Schwochau, 1970). The synthetic peptides poly-L-lysine and poly-L-arginine are elicitors but are unlikely to be *natural* pisatin elicitors. However, these basic peptides provide clues regarding the potential of natural protein segments rich in arginine or lysine to act in this capacity (Brunner et al., 2002). These synthetic proteins (peptides) can be mimicked by carbohydrates that are also strongly positively charged. Chitosan is a basic polymer of glucosamine and is a signaling component in the pea/Fusarium interaction (Hadwiger et al., 1981; Hadwiger, 2015b). Chitosan shares the DNA affinity property of basic peptides and can activate the same responses in pea endocarp tissue as the bean pathogen, *F. solani* f. sp. *phaseoli* (Fsph) (Loschke et al., 1983). Furthermore, a large group of microbes contain chitin (polymers of β-linked *N*-acetyl glucosamine) and chitosan (polymers of β linked glucosamine). Chitosan heptamers of seven sugars or more represent optimal-sized elicitors (Kendra et al., 1989). Although chitin structure has similarities to chitosan, the added acetyl group negates the positive charge of the amino groups, rendering it less effective as an elicitor unless there is a chitin receptor to carry forth the signal (Hadwiger and Chang, 2015). A computer analysis indicates that the chitosan heptamer (seven glucosamine residues) fits into the minor groove of the DNA molecule (Hadwiger et al., 1989).

## Other Minor Groove Targeting Agents

**FIGURE 6:**
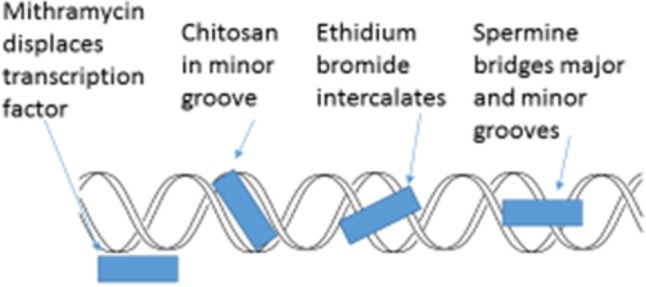
Schematic representation of four major actions on the helical condition of DNA. Mithramycin as a minor groove targeting agent displaces the Sp2 transcription factor (Neigeborn and Carlson, 1984; Miskovic et al., 2013). Chitosan resides in the DNA minor groove (Hadwiger et al., 1989). Ethidium bromide intercalates between DNA base pairs (Lenglet and David-Cordonnier, 2010). Spermine can both enter the minor groove and the major groove in a manner that enables the bridging of both (Bryson and Greenall, 2000).

**FIGURE 7:**
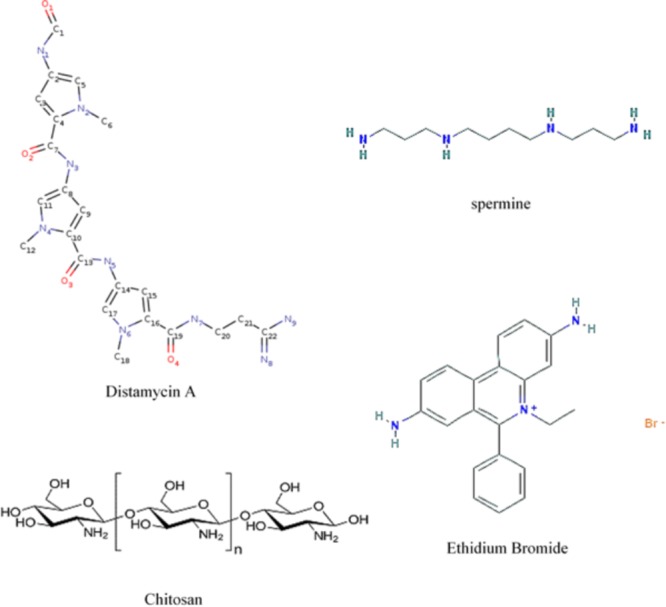
Formulas showing molecular locations of nitrogens when present and planar ring structures. Distamycin A, spermine, and ethidium bromide (by permission from NIH – PubChem and Cayman Chemical) and chitosan.

**FIGURE 8:**
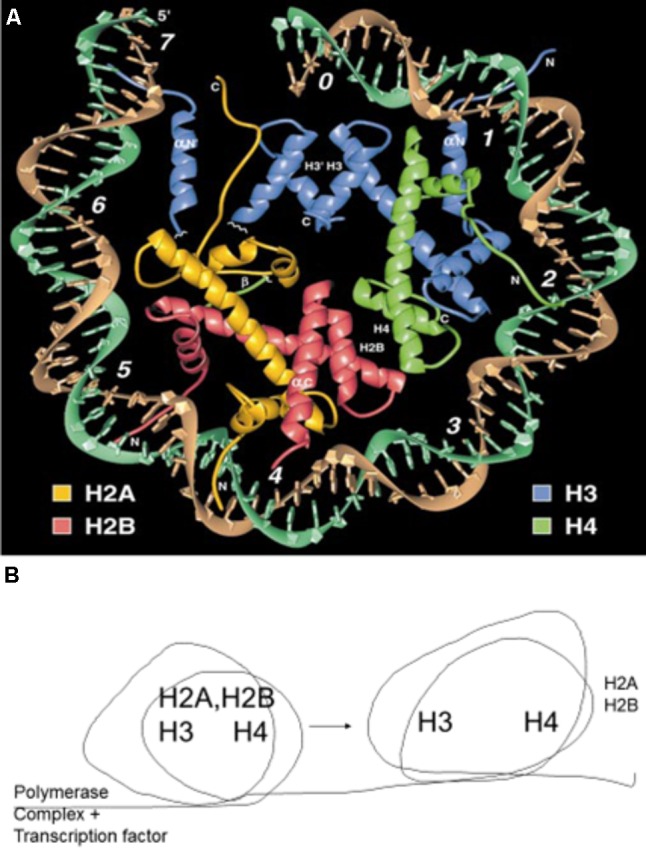
**(A)** Crystal structure of a nucleosome. The DNA helix circles the attached histones, H2A, H2B, H3, and H4. Reproduced with copyright permission (Luger et al., 1997). **(B)** Schematic description of the nucleosome rolling action that results in the temporary removal of histones H2A and H2B (Gerasimova et al., 2016). This temporary removal is essential for the RNA polymerase complex to transcribe genes and the histones are reassembled following the successful passing of the complex.

The minor groove of DNA is a target of anticancer drugs (**Figure 6**). These include distamycin A and mithramycin (**Figure 7**), and Hoechst 33258 (pibenzimol), 4′,6-diamidino-2-phenylindole (DAPI) and neptropsin, which are topoisomerase poisons or helicase inhibitors, preferring an AT-tract duplex DNA (Varqiu et al., 2008). Chitosan has had only limited evaluations as an anticancer agent; however, chitosan, actinomycin D, and camptothecin all activate the production of p53, a tumor-suppressing protein, in the mouse pre-neoplastic mammary cell line CL-S1 (Hadwiger et al., 1997). All three agents activate defense genes in pea (Isaac et al., 2009), and although each agent is capable of altering chromatin structure within the nucleosome (**Figure 8**), they reportedly have differing specific modes of action. The concentration of positive charges on chitosan may compete with pea histones that function to compact the cellular DNA in the nucleus (Hadwiger, 2008; Isaac et al., 2009). Chitosan’s action on chromatin is able to loosen the compaction of the nucleosome structure, allowing stalled genes to resume transcription (Hadwiger, 2015a). Messenger RNA from chitosan-treated pea tissue when transcribed in an *in vitro protein synthesis system* also produces protein 2-D patterns closely related to mRNA from pea tissue that responds to a bean pathogen, *Fusarium solani* f. sp. *phaseoli* (Fsph) (Loschke et al., 1983).

## DNA Groove-Binding Architectural Proteins

Chromatin architectural proteins is a major group of nuclear proteins that impact chromatin structure and function. TATA-box-containing protein and high mobility group HMG A protein complexes with DNA can have sequence-specific recognition (Bewley et al., 1998; Klosterman and Hadwiger, 2002). Both bind in the minor groove of DNA and make conformational changes in the DNA. Both occur widely in eukaryotic organisms, including plants. Some chemicals that binds the minor groove of DNA such the bis-benzimidazoles (Hoechst 33258) and DAPI have been used for cancer therapy (Baraldi et al., 2004). These compounds interact physically with DNA and cause reversible inhibition of DNA-dependent functions. Hoechst 33258 (but not DAPI) was found to be an elicitor (weak) of phytoalexin production in pea tissue. These two compounds, along with chitosan, reportedly enter the DNA minor groove (Baraldi et al., 2004). The strong induction by the chitosan preparation (**Table 1**) with heptamer-sized polymers may benefit from molecular lengths large enough to outperform the smaller spermine and Hoechst 33258 compounds in initiating the pisatin induction, using this single parameter for comparison. Given information on the mechanism of action of DNA-specific *abiotic* compounds in altering the DNA within chromatin and activating a defense response, the information should be useful to understand the mechanism of the *biotic* DNA-specific action of chitosan that also occurs in the minor groove of DNA. Similarly, a comparison of the multiple compounds utilized in cancer therapy would be useful in determining which groove-binding abiotic compound was most active or inactive in inducing collateral gene activation responses. The pisatin assay was utilized to evaluate the optimal accumulations that could be generated by the compounds listed in **Table 2**.

In calf thymus tissue, the non-histone proteins HMG 1 and HMG 2 are capable of unwinding the DNA double helix. Pea tissue also possesses a HMG A protein (Klosterman et al., 2003) that is reduced in the chromatin material during the initiation of the pea defense response (Isaac et al., 2009). HMG A is considered an architectural transcription factor with a wide array of actions in both stabilizing and altering chromatin structure. Its action is reportedly influenced by the associated salt solution of the assay (Javaherian and Sadeghi, 1979).

There is an alteration of nuclear structure that occurs in the early minutes of pea/*Fusarium solani* formae species interactions. Interactions at 5 h were globally more intense in the compatible interaction than in the resistance reaction (Isaac et al., 2009). Western analyses, mass spectrometry, and [^32^P] techniques were used to follow the disappearance of the architectural transcription factor HMG A and histones H2A/H2B. Of more specific interest, at 5 h, these nuclear proteins were also observed to be less abundantly complexed in the vicinity of two PR genes, DRR206 and the β-glucanase gene, utilizing chromatin immunoprecipitation analyses. There is an early ubiquitination of HMG A and some histones (Isaac et al., 2009). This suggests that the DNA breaks and the removal of nuclear proteins may assist the progression of stalled genes that had previously been obstructed. Some of the specific defense genes become activated as nuclear proteins (histone/HMG A) are removed.

## DNA Damage and Repair Aspects From Cancer Therapeutic Research

DNA damage is an early event the pea endocarp/fungal pathogen interaction (Tanaka and Hadwiger, 2017) and occurs following other DNA inducing treatments. DNA damage is also a linking mechanism in animal immunity development (Brzostek-Racine et al., 2011; Nakad and Schumacher, 2016). The DNA damage activates immune signaling through molecular and cellular pathways and drives chronic inflammation in humans. The DNA damage response can also induce interferon production.

Some of the chemotherapy-induced DNA damage responses include genes for DNA repair (Woods and Turchi, 2013). Ataxia telangiectasia mutated (ATM) kinases are activated and phosphorylate many substrates, including proteins involved in checkpoint activation, DNA replication and DNA damage repair. It remains uncertain whether ATM binds directly to DNA. As indicated above, the transcription complex movement is facilitated in the absence of helical blocks and histone attachments to the DNA. Additional effects on the helical structure are possible by non-histone proteins such as HMG A (Isaac et al., 2009), which can unwind the double helix, and by nucleases such as ribonuclease and gyrases, which can cause destabilizing effects on DNA helical structure (Felsenfeld et al., 1963). The development of anticancer drugs needs to take these direct actions on DNA into consideration. Alternately, the defense response of pea tissue is strongly affected by these additional proteins. Ribonuclease A strongly induces the accumulation of the pea phytoalexin pisatin. This activity is diminished by half if the ribonuclease is autoclaved prior to application, and reportedly, ribonuclease S loses half of its pisatin-inducing potential if only the non-enzymatic portion of the “S” molecule is applied, indicating that the action is a combination of enzyme activity and non-enzymatic cationic proteins. Additionally, the digestion of the RNA content may have a functional role in chromatin structural change. A number of other basic proteins also induce pisatin production without any obvious nuclease activity (Hadwiger et al., 1974).

## Reactive Oxygen Species (ROS): Potential Signals in Cancer and Disease Resistance Via DNA Damage

Reactive oxygen species (ROS) production is a mechanism shared by all non-surgical therapeutic approaches for cancers, including chemotherapy radiotherapy and photodynamic therapy. ROS are usually increased in cancer cells due to oncogene activation and are involved in the initiation, progression and metastasis of cancers. Thus, ROS are considered oncogenic (Wang and Yi, 2008). Oxidative stress has a significant impact on the progression of cancer and other human pathologies. It has a global influence on chromatin structure, mediating a number of cellular changes, including gene expression. This makes the targeting of oxidative stress pathways important in the control of cancer (Kreuz and Fischle, 2016). ROS in eukaryotic tissue cause multiple DNA base changes, such as from thymine to thymine glycol (Dizdaroglu and Jaruga, 2012) and 5-hydroxy methyl-2-deoxy uridine (Chaung and Boorstein, 1997). Most of these changes cause mismatches during DNA replication, leading to mutagenesis. ROS are capable of directly altering plant DNA. Application of hydrogen peroxide to pea endocarp tissue increases DNA fragmentation and activates defense genes (PR genes) (Tanaka and Hadwiger, 2017). Although direct effects on the DNA are detectable in pea, it is likely that other damage to the pea chromatin is involved and that the induction of repair responses may occur as it does in animals. ROS mediate a systemic signal network for developing plant immunity (Alvarez et al., 1998). A part of this network is the DNA damage inflicted by ROS. In pea tissue, this damage is associated with the post-treatment period during which PR genes are activated (Tanaka and Hadwiger, 2017).

Reactive oxygen species are induced in mammalian tissue as an antimicrobial defense. Their importance is based on the observation that individuals with deficiencies in generating ROS are highly susceptible to infection by a broad range of microbes. A likely mode of defense occurs following damage to mitochondrial DNA. Interestingly, DNA repair mechanisms were required to resist killing by ROS. Although ROS play a role, direct killing may not be the key mechanism. ROS may affect ROS-dependent signaling controls, such cytokine production (Deffert et al., 2014). Excessive ROS can damage cellular proteins, lipids and DNA, leading to fatal lesions in cells that contribute to carcinogenesis. Low levels of ROS facilitate cancer cell survival. High levels of ROS can suppress tumor growth through the sustained activation of cell-cycle inhibitors and the induction of cell death (Ramsey and Sharpless, 2006). A cancer cell can die in three ways: apoptosis, necrosis and autophagy. The cytotoxic nature of ROS is the driving force behind apoptosis, but with even higher amounts, ROS can result in both apoptosis and necrosis, a form of uncontrolled cell death in cancer cells (Hampton and Orrenius, 1997).

## Other Potential Signals in Disease Resistance Via DNA Damage

It has been reported other potential signals for nonhost disease resistance via DNA damage as shown in **Figure 1** (Yan et al., 2013; Hadwiger and Tanaka, 2015, 2017b). Another potential signal for speculation is damage-associated molecular patterns (DAMPs; Tanaka et al., 2014). For examples, extracellular DNA that trigger plant immunity are in addition to the hypothesized PAMPs discussed above. These signals are unique in that only self-DNA fragments (Barbero et al., 2016; Duran-Flores and Heil, 2018) and are active and maybe differ from the PAMPs in that the likely target is host DNA rather than a pattern recognition receptor located in the vicinity of the cell membrane. The specificity of the self-DNA requirement and the rapidity of the response were demonstrated by developing immunity in common bean with extracellular DNA from other same species plants and unsuccessfully from DNA from unrelated species. The fragments all less than 700 bp suggest that the molecules reach host DNA and that like other introduced DNA can quickly find homologous regions in the genome. The mechanism of action has not been unequivocally determined (Mazzoleni et al., 2015a,b) but the presence of a homologous third strand fragment is likely destructive or at least competitive to chromatin organization in the homologous region. As indicated previously seemingly minor changes in chromatin organization can effect transcription. Interestingly, these externally applied DNAs can also affect plant growth.

## Essence of the Compiled Information on DNA Damage in Disease Resistance in Plants and Cancer Development in Animals

Multiple black boxes of unknown regulatory components are prevalent within chromatin (Dekker et al., 2013). Such variations in chromatin structures were demonstrated in the 1950s by a cytologist looking at the bands within giant chromosomes of *Drosophila* salivary glands. Regions sensitive to treatments with hormones, DNA intercalators, heat, etc. were observed to puff out from certain bands of the chromatin within the giant chromosome. The multi-action damage of ROS to plant nuclei activating defense responses may be more global in comparison to activation by actinomycin D, which recognizes specific DNA sequences. This actinomycin-DNA specificity results in more direct action on sensitive areas of the chromatin (Lewis et al., 1975), as visualized in the puffing effects on giant chromosomes of *Drosophila* (Watson et al., 1987). In general, actinomycin D prefers GpC regions. It binds to DNA by intercalating its phenoxazine ring at a GpC step such that the two cyclic pentapeptides of the drug area are located in the DNA minor groove (Sobell and Jain, 1972). As the base sequence becomes more deviant, there can be more radical changes. For example, actinomycin D induces nucleotide flipping out, sharp bends and a left-handed twist in CGG triplet repeats. Heat denaturation, circular dichroism and surface plasmon resonance analyses indicate that adjacent GpC sequences flanking a G:G mismatch are preferred actinomycin D binding sites (Lo et al., 2013). The detection of sensitive regions within chromatin regions of pea chromosomes has been defined genetically as QTLs. The mapping of these regions in pea detects some of the induced defense genes residing within QTLs and thus may characterize special features of the pea chromosome (Pilet-Nayel et al., 2002).

## Condensed State of DNA in Plant Chromatin

Nucleosomes help condense the almost 1-m length of DNA within a pea cell, and similarly in many other eukaryotic cells, into the small volume of the nucleus of ∼10 microns in diameter (Hadwiger and Adams, 1978). This fete is accomplished in part in co-operation with nucleosomes. Each turn of the nuclear DNA strand may contain six nucleosomes, as shown in the **Figure 8A**, each composed of two molecules of the following histone molecules: H3, H4, H2A, and H2B. This structure is stable because of the electrostatic interaction between the negatively charged DNA and the basic histones (Yaniv, 2014).

The presence of histones and the condensed structure of chromatin restricts the access of specific proteins to DNA sequences except when appropriate for transcription, repair, etc. (Petesch and Lis, 2008; Pang et al., 2013; Mao et al., 2014). Both RNA and DNA polymerases must separate the strands of DNA. This can be accomplished enzymatically in eukaryotic cells by helicases. Helicase motifs have been found in genetic complexes (SNF genes in yeast that act in control of certain other genes) (Neigeborn and Carlson, 1984). Re-expression of the helicase in human cells previously lacking a helicase strongly increased the expression of a glucocorticoid hormone receptor (GR) (Muchardt and Yaniv, 1993). These examples suggest a helicase function in DNA strand separation that assists the transcription process.

The broad implication of complexes containing SNF genes in cancer is that the loss or change in this activity can result in a multitude of re-regulated cellular programs affecting cell survival and cell death of malignant transformation and may relate to the strand separating function of helicases. In plants, the re-regulation of genes due to abnormal insults to organized chromatin via pathogen invasion can also affect transcription patterns for cell viability and cell death. Fortunately, there are windows in this array in which previously suppressed genes can become beneficial to the immune response of the plant tissue.

The signaling of such new gene expression levels with respect to time after inoculation of an “inappropriate” pathogen has been defined as what is termed a nonhost resistance response (Hadwiger, 2015c). This early response is almost universally observed as more excessive than the plant’s response to pathogens considered to be in the range of that particular plant species. Plants have obviously diverted evolutionarily from animal systems; however, there is a conservation of similarity in the transcriptional machinery within plant and animal cells. It was recognized early that the amino acid sequences of an array of histone proteins in both plant and animal cells were highly conserved. Additionally, there is some similarity in certain transcription factors such as HMG A, which is regarded as an architectural transcription factor with AT-hook motives within the protein specific to AT-rich regions of the DNA (Klosterman et al., 2000; Klosterman et al., 2003; Reeves, 2010). HMG A is retained in both plant and animal systems. Plants and animals also have SNF/SWI-like complexes that affect transcription (Bezhani et al., 2007; Jerzmanowski, 2007; Sarnowska et al., 2016).

A portion of the pea and cancer cell chromatin contain genes with various states of activity ranging from open expression to “stalled” (Nelson et al., 2007). Stalled genes have obstructions that are related to the state of transcription factors, DNA helical obstructions and nuclear protein content. Therefore, it is likely that chromatin modifications from agents with slightly different modes of action can assume multiple changes increasing (or suppressing) gene expression. Furthermore, because the agents can possess differing base-sequence preferences, their proximity to the genes expressed will also be an influencing factor. Conformational states of chromatin in the vicinity of the PR genes may effect transcription enhancement via nucleosome disassembly or histone H2A/H2B releases similar to that found in other eukaryotic systems (Adkins and Tyler, 2006; Weake and Workman, 2008).

In plant cells, the production of pisatin can occur by the amplification of a secondary metabolic pathway that depends on increases in one or more enzymes. The induction of pisatin is usually in synchronization with the activity of PR genes, and both entities possess anti-fungal properties, thus implicating regulatory enhancements in a group of plant genes. The same DNA-specific agents confront similar chromatin structures in animal cancer cells, but the medicinal objectives are intended to negatively affect the viability of actively dividing cancer cells. These negative properties of DNA-specific agents are often acquired with high agent concentrations, it is inevitable that non-targeted peripheral areas will receive diluted concentrations. The results obtained in plant tissue suggest that there is the potential for the lower concentrations of anti-cancer agents to cause a different array of effects.

## Perspective Summarization

**FIGURE 9:**
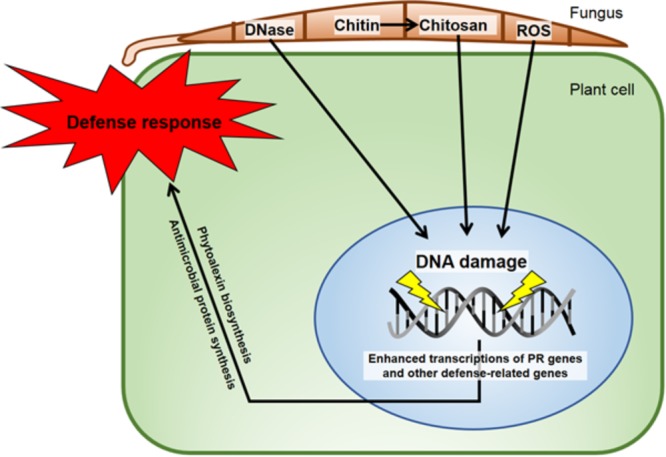
Diagram of some DNA-direct effects of biological entities shown capable of activating disease resistance responses in plants. The minor groove-localizing chitosan heptamer is released from the fungus by the plant chitinase and the fungal chitin deacetylase enzymes (Kendra et al., 1989). The DNA torsion/helicity can be affected by small molecule binding/intercalation and single strand cleavage by *Fusarium solani* f. sp. *phaseoli* (Fsph)-DNase (Hadwiger and Polashock, 2013). ROS released in the interaction (Tanaka and Hadwiger, 2017) have multiple ways to alter nuclear DNA. The isoflavonoid phytoalexins, e.g., pisatin (Hadwiger, 2015a), and defensin (Almeida et al., 2006) are directly anti-fungal. Fsph DNase accumulation within Fsph terminates fungal growth (Hadwiger, 2015c). PR genes such as DRR49 codes for RNase and DRR230 codes for defensin. Chitinase and β-glucanase that digest fungal chitin and glucan polymers, respectively (Kendra et al., 1989) are present in healthy tissue and increase following fungal challenge. All proteins that transient the host–parasite interface are produced with a N-terminal signal peptide for transfer through membranes (Hadwiger, 2009).

Following DNA damage within human or plant cells, there is an alteration of the repressed states of some genes encumbered within the respective nuclei. The damage results in actions, such as defense gene activation in plants and suppression of growth in cancer cells with eventual side effects, including programmed cell death (apoptosis). Many of the effects can be duplicated by targeting nuclear DNA by eliciting agents with varying modes of action, such as through DNA intercalation, DNA cleavage, base substitution, nuclear protein modification, etc., that elicit varying responses. This targeting of the sensitive chromatin regions by chemically different agents can produce similar transcriptional changes to those in real biological systems. This *abiotic* probing provides insight into the *biotic* changes (**Figure 9**) experienced by the nucleosomes of the nuclear chromatin of both plant and animal cells. Because of the highly conserved components of chromatin in plants and animal cells, the mechanisms of these changes can have implications that are useful in understanding both systems.

Transcription data over the decades have implicated DNA torsional changes as central to the progression of RNA polymerase complexes through gene open reading frames (Ma et al., 2013). These enhancements of newly expressed genes must remove the barricades of helical stress and nucleosome condensation that restricts the ORF read through RNA polymerase and the subsequent expression of defense and DNA repair genes. A DNA-intercalating scenario may be to insert into proximal DNA, reversing the negatively supercoiled or dispersing nucleosome structure. Another action may be the ubiquitination of histones H2A/H2B and removal from the area downstream from the RNA polymerase complex (**Figure 8B**). The enhancement of the defense responses in plants can occur in a similar manner. In the latter case, it is the components of the response, the antifungal compounds, that enable resistance. In some plant/bacterial interactions, the complete killing of cells surrounding the lesion is beneficial to resistance as well. The lesson available from the plant responses for cancer therapy is that elicitor-initiated gene activations occurring with low-level treatments in plants may occur randomly at the fringes of the high-level anticancer treatments and may activate genes associated with adverse side effects.

The plant responses that develop from the large number of eliciting agents tested on pea endocarp tissue (**Figures 1, Figures 9**) indicate that cellular chromatin structural changes relate to the presented chemistry of the agent without respect for what a pharmaceutical company designates as the cellular target. That is, the agent may be designated an antimalarial, antidepressant drug, etc.; however, if there are potential intercalating rings and positive charges exposed, the agent will likely localize next to the negative charges of the DNA and the resulting transcriptional changes will occur based on the chemistry of the interaction.

## Author Contributions

LH and KT wrote the manuscript.

## Conflict of Interest Statement

The authors declare that the research was conducted in the absence of any commercial or financial relationships that could be construed as a potential conflict of interest.
